# Chilaiditi Syndrome With a Large Colonic Loop in a Patient With Autonomic Nervous System Dysfunction

**DOI:** 10.7759/cureus.15877

**Published:** 2021-06-23

**Authors:** Christos Sotiropoulos, Eftichia Sakka, Georgia Diamantopoulou, Georgios Theocharis, Konstantinos Thomopoulos

**Affiliations:** 1 Gastroenterology, University General Hospital of Patra, Patra, GRC; 2 Internal Medicine, General Hospital of Patra "St Andrew", Patra, GRC

**Keywords:** chilaiditi syndrome, chilaiditi sign, bowel interposition, pneumoperitomeum, autonomic nervous system dysfunction

## Abstract

Chilaiditi syndrome is an unusual condition in which radiographic evidence of a large bowel interposition between the liver and the right hemidiaphragm appears in the chest X-ray. The etiology is unknown and the clinical symptoms vary from case to case. The special characteristics of the syndrome can easily lead to a misdiagnosis and a CT scan is needed to avoid surgical interventions for a suspected pneumoperitoneum. We present a 48-year-old female patient with a medical history of autonomic nervous system dysfunction who referred to the Emergency Department (ED) due to abdominal pain. Chest radiography revealed a radiographic sign of pneumoperitoneum but a CT scan of the abdomen showed interposition of the right colon in the right hemithorax between the diaphragm and the liver without any signs of perforation. The patient was treated with bowel decompression and her symptoms resolved gradually. So far, there is no other case of Chilaiditi syndrome in a patient with autonomic nervous system dysfunction in the published literature. To conclude, Chilaiditi’s sign is an unusual radiographic sign presenting as a pneumoperitoneum in the chest X-ray. In order to avoid misdiagnosis and unnecessary surgical interventions, a CT scan should be ordered.

## Introduction

Chilaiditi syndrome is a rare medical condition in which there is radiographic evidence of a large bowel interposition between the liver and the right hemidiaphragm [1-5]. The estimated incidence of Chilaiditi’s sign ranges from 0.025% to 0.28% and appears more frequently in men with a median age of 60 [1-6]. It can be congenital or it can be developed in patients with obesity, liver cirrhosis, or right diaphragm paralysis [1-3]. The colonic interposition results in various clinical manifestations [1-4]. It can be asymptomatic or symptomatic causing abdominal or chest pain, nausea and vomiting, bloating and constipation [1-4]. The rarity of the disease and the variation in the clinical presentation can lead to a misdiagnosis, as it can be easily mistaken as pneumoperitoneum due to perforation [1-2]. Awareness of this condition and its consideration as a differential diagnosis is crucial to prevent unnecessary interventions [3].

## Case presentation

A 48-year-old female patient was referred to the Emergency Department (ED) due to a three-day history of abdominal pain, nausea, constipation, dizziness, and orthostatic hypotension.

Her past medical history included an autonomic nervous system dysfunction of unknown cause, diagnosed at the age of 22, where she developed dysautonomia with dizziness, orthostatic hypotension, difficulty swallowing, constipation, sweating abnormalities, and blurry vision. She was treated periodically by lifestyle changes (head elevation of the bed, slow position change, fluid supplementation, salty food), compression stockings, feeding tubes, and prescription medication (sympathomimetic agents, dopamine-blocking agents, laxatives, prokinetic agents).

**Figure 1:**
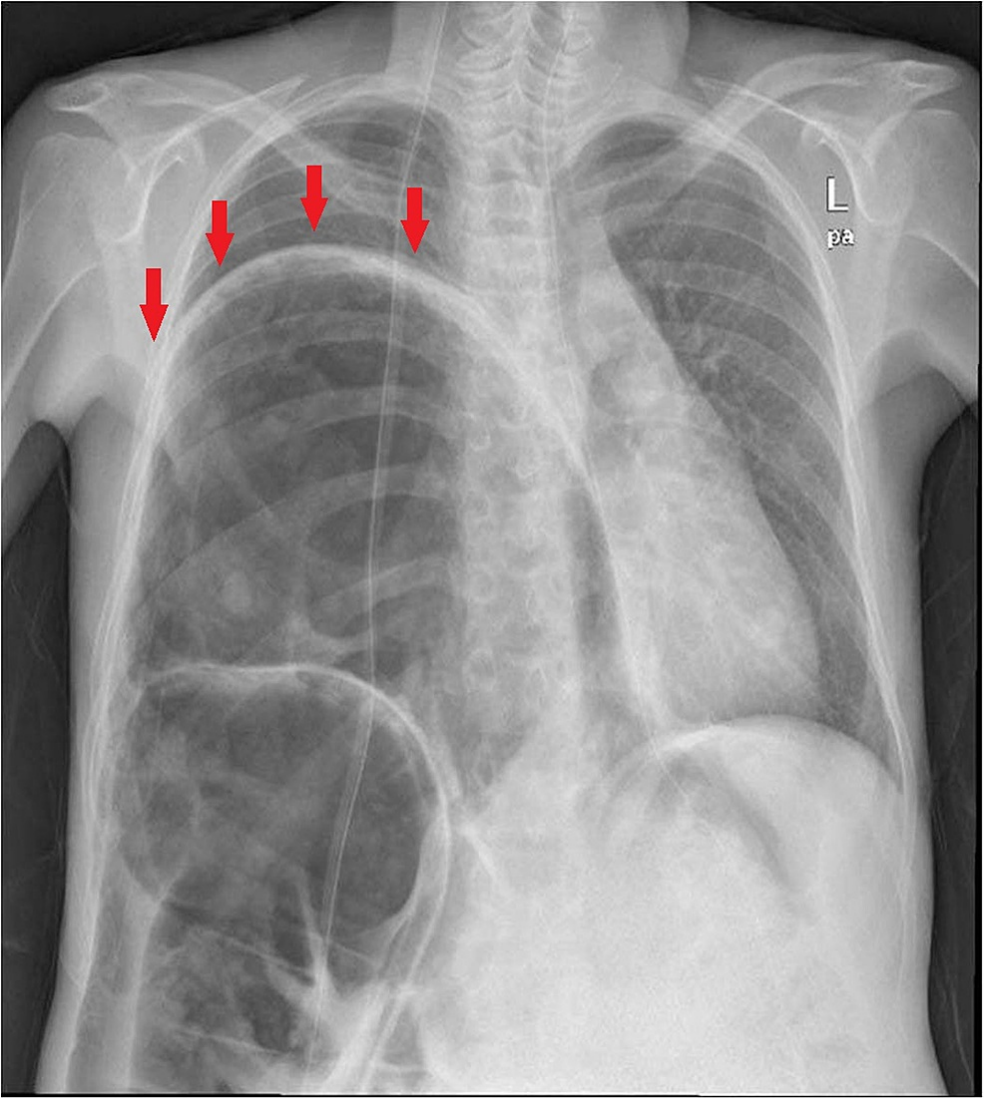
Chest radiography shows an excessive right diaphragmatic eventration and a distended bowel filled with gases under the right hemidiaphragm (red arrows).

On physical examination there was tenderness in the right upper quadrant with abdomen distention; there was no rebound tenderness or guarding and the bowel sounds were normal. Routine laboratory tests including electrolytes, complete blood count, and hepatic panel were normal, but posteroanterior chest radiography revealed a right diaphragmatic eventration with free gas below (Figure 1).

**Figure 2:**
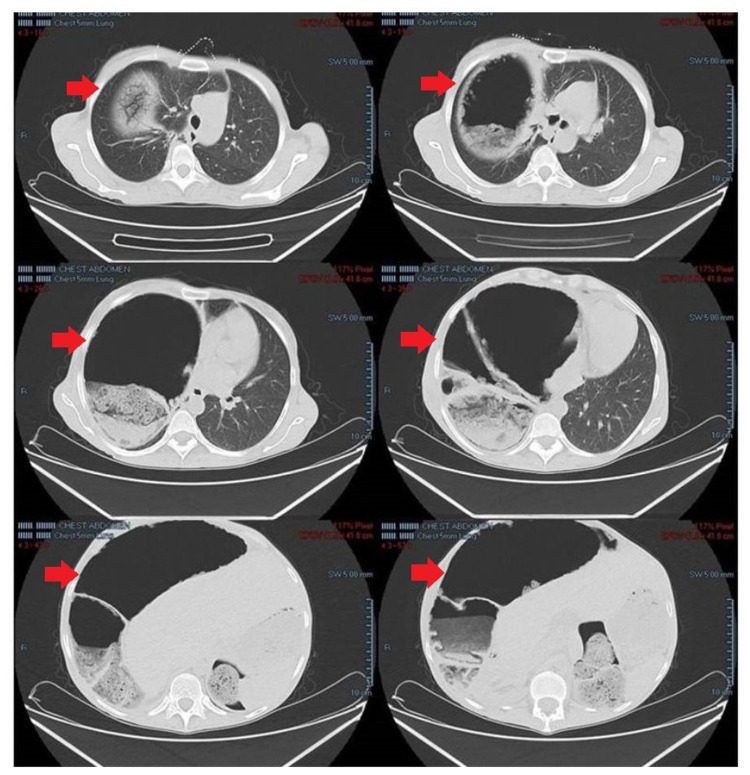
Abdominal CT shows the interposition of the right colon in the right hemithorax between the diaphragm and the liver without any signs of perforation (red arrows).

A pneumoperitoneum was suspected and a CT scan of the abdomen was immediately ordered. The CT scan showed an interposition of the right colon in the right hemithorax between the diaphragm and the liver without any signs of perforation (Figure 2).

As a result, the patient was diagnosed with Chilaiditi syndrome. Our patient was treated with nasogastric decompression, bowel decompression via colonoscopy and bowel rest and her symptoms resolved gradually.

## Discussion

Chilaiditi syndrome took its name from a Greek radiologist, Demetrious Chilaiditi, who first described the radiographic sign in 1911 [1-2, 6-7]. Chilaiditi’s sign is the result of the malposition of the colon between the diaphragm and the liver [1-5]. This phenomenon indicates the manipulation of any of these three organs (diaphragm, liver, colon) which can predispose to such organ misplacement [3]. The diaphragmatic causes include right-sided diaphragmatic hemiparesis (phrenic nerve damage) and diaphragmatic eventration (congenital loss of the muscular fibers of the diaphragm) [3]. Potential hepatic causes are the loss of tone in the falciform ligament and a small liver secondary to cirrhosis or hepatectomy [3]. Last but not least, dolichocolon, ascites, and obesity are some other predisposing factors associated with increased risk of developing Chilaiditi’s sign [3].

The presentation can significantly vary and primarily the diagnosis is made through radiographic evaluation with CT scan [1-4]. It can be asymptomatic and is mostly diagnosed as an incidental finding (Chilaiditi’s sign) [1-4]. The characteristic radiographic finding is air below the diaphragm that does not change with the position change of the patient [1-2]. However, symptoms ranging from nonspecific intermittent mild abdominal pain to acute intestinal volvulus are also reported [1-4].

Autonomic nervous system dysfunction can affect several basic functions, including heart rate, body temperature, breathing rate, digestion, and sensation. Among others, it may cause colonic pseudo-obstruction with distention and interposition of the colon that may lead to Chilaiditi’s sign. Asymptomatic patients do not need any intervention, while symptomatic need bowel rest, fluid supplementation, bowel decompression, cathartics, a high-fiber diet, and stool softeners [1-2, 4, 7]. Surgical treatment is needed only in complicated cases with ischemia or perforation [1-2].

## Conclusions

Chilaiditi’s sign is an unusual radiographic sign, but it must be suspected in a chest X-ray that reveals an image compatible with pneumoperitoneum. In order to avoid misdiagnosis and unnecessary surgical interventions, a CT scan, the gold-standard diagnostic tool for Chilaiditi’s sign, should be ordered. This case highlights the significance of clinical awareness of this syndrome among physicians to reduce the need for unnecessary surgical interventions.
